# Disrupted default mode network connectivity in migraine without aura

**DOI:** 10.1186/1129-2377-14-89

**Published:** 2013-11-08

**Authors:** Alessandro Tessitore, Antonio Russo, Alfonso Giordano, Francesca Conte, Daniele Corbo, Manuela De Stefano, Sossio Cirillo, Mario Cirillo, Fabrizio Esposito, Gioacchino Tedeschi

**Affiliations:** 1 Department of Neurology, Second University of Naples, Piazza Miraglia 2 - I-80138, Naples, Italy; 2 Institute for Diagnosis and Care “Hermitage Capodimonte”, Naples, Italy; 3 Neuroradiology Service, Second University of Naples, Naples, Italy; 4 Department of Cognitive Neuroscience, Maastricht University, Maastricht, The Netherlands; 5 Department of Medicine and Surgery, University of Salerno, Salerno, Italy

**Keywords:** Resting-state fMRI, Default mode network, Migraine

## Abstract

**Background:**

Resting-state functional magnetic resonance imaging (RS-fMRI) has demonstrated disrupted default mode network (DMN) connectivity in a number of pain conditions, including migraine. However, the significance of altered resting-state brain functional connectivity in migraine is still unknown. The present study is aimed to explore DMN functional connectivity in patients with migraine without aura (MwoA) and investigate its clinical significance.

**Methods:**

To calculate and compare the resting-state functional connectivity of the DMN in 20 patients with MwoA, during the interictal period, and 20 gender- and age-matched HC, Brain Voyager QX was used. Voxel-based morphometry was used to assess whether between-group differences in DMN functional connectivity were related to structural differences. Secondary analyses explored associations between DMN functional connectivity, clinical and neuropsychological features of migraineurs.

**Results:**

In comparison to HC, patients with MwoA showed decreased connectivity in prefrontal and temporal regions of the DMN. Functional abnormalities were unrelated to detectable structural abnormalities or clinical and neuropsychological features of migraineurs.

**Conclusions:**

Our study provides further evidence of disrupted DMN connectivity in patients with MwoA. We hypothesize that a DMN dysfunction may be related to behavioural processes such as a maladaptive response to stress which seems to characterize patients with migraine.

## Background

Migraine is a common and disabling primary headache disorder clinically characterized by episodic attacks of throbbing headache with specific features and associated symptoms [1]. A great body of studies has been conducted on patients with migraine, however, its pathophysiology is not completely understood. Indeed, currently, no integrative model has been formulated that accounts for all the factors that may play a role in migraine pathophysiology such as spreading depression, neurogenic inflammation, excitatory/inhibitory balance, genetic background and disturbed energy metabolism [2,3]. More recently, converging evidence supports the role of a maladaptive stress response in migraine mechanisms [4,5]. Resting-state fMRI (RS-fMRI) has allowed for the exploration of brain connectivity between functionally linked cortical regions, the so-called resting-state networks (RSNs) [6]. The most consistently reported RSN is the default mode network (DMN), which plays a relevant role in adaptive behavior other than in cognitive, emotional, and attention processes [7,8]. In the last years, several RS-fMRI studies have identified functional connectivity changes in patients with migraine without aura (MwoA) [9]. To our knowledge, only a few of them have focused on DMN integrity in patients with migraine, reporting inconsistent results [10-12]. For this reason, we investigated the DMN connectivity in patients with MwoA during the interictal period, by means of RS-fMRI using an independent component approach (ICA) to avoid any a priori hypothesis about the source of a possible functional disconnection [13]. In addition, we used Voxel Based Morphometry (VBM) to assess whether any between-group differences in resting-state functional connectivity were dependent on structural abnormalities, recently described in patients with migraine [14]. We hypothesized that DMN connectivity could be decreased in patients with MwoA in comparison to healthy controls (HC), supporting the current view that migraine may be related to a maladaptive stress response.

## Methods

### Patients

Twenty-five consecutive patients with episodic MwoA, according to the International Headache Society criteria (Headache Classification Subcommittee of the International Headache Society, 2013) [15] were prospectively recruited from the migraine population referring to the outpatient headache clinic of the Department of Neurology at the Second University of Naples. Demographic data and the following clinical characteristics were obtained from the patients with MwoA: age of onset, disease duration, frequency (day/month), duration and mean pain intensity of migraine attacks and related disability. Mean pain intensity of migraine attacks was assessed using a visual analogic scale (VAS). To obtain an accurate assessment of patient’s headache-related disability, all patients with MwoA completed the Migraine Disability Assessment Scale (MIDAS) and Headache Impact Test (HIT-6). Patients with hypertension, diabetes mellitus, heart disease, other chronic systemic diseases, stroke, cognitive impairment, substance abuse, chronic pain, as well as other neurological or psychiatric disorders were excluded. To avoid any possible migraine attack-related or pharmacologic interference with the RS-fMRI investigation, all patients with MwoA were both migraine-free and not taking attack medications for at least 3 days before scanning and were naïve for any commonly prescribed medications for migraine prevention. Moreover, all patients with MwoA were interviewed 7 days after scanning to ascertain if they were migraine-free also during the post-scan week. For this reason, five patients were excluded from the analyses herein, which focused on 20 right-handed patients (mean age ± SE: 28.15 ± 3.08 years, 10/10 males/females).

### Healthy controls

Twenty age- and gender-matched, right-handed subjects (mean age ± SE: 28.90 ± 3.63 years, 10/10 females/males) with less than a few spontaneous non-throbbing headaches per year, with no family history of migraine, no hypertension, diabetes mellitus, heart disease, other chronic systemic diseases, stroke, cognitive impairment, substance abuse, chronic pain, as well as other neurological or psychiatric disorders were recruited as HC.

### Standard protocol approvals, registrations, and patient consents

The study was approved by the Ethics Committee of Second University of Naples, and written informed consent was obtained from all subjects according to the Declaration of Helsinki.

### Neuropsychological evaluation

To assess levels of depression and anxiety, patients with MwoA and HC completed the Hamilton Depression Rating Scale (HDRS) and the Hamilton Anxiety Rating Scale (HARS). An extensive neuropsychological evaluation was performed in patients with MwoA as previously described [16].

### Imaging parameters

MRI was performed on a General Electric (Minneapolis, U.S.) Signa HDxt 3 Tesla whole-body scanner equipped with an 8-channel parallel head coil. RS-fMRI data consisted of 240 volumes of a repeated gradient-echo echo planar imaging T2*-weighted sequence (TR = 1508 ms, axial slices = 29, matrix = 64 × 64, field of view = 256 mm, thickness = 4 mm, interslice gap = 0 mm, 10 discarded scans at the beginning). During the functional scan, subjects were asked to simply stay motionless, awake, and relaxed, and to keep their eyes closed; no visual or auditory stimuli were presented at any time during functional scanning. Three-dimensional high-resolution T1-weighted sagittal images (GE sequence IR-FSPGR, TR = 6988 ms, TI = 1100 ms, TE = 3.9 ms, flip angle = 10, voxel size = 1 × 1 × 1.2 mm3) were acquired for registration and normalization of the functional images as well as for atrophy measures and VBM analysis.

### Statistical analysis of clinical data

Demographic and clinical features of patients with MwoA and HC were compared by the t-test for independent samples or by χ2, as appropriate.

### RS-fMRI pre-processing and statistical analysis

Image data pre-processing and statistical analysis were performed with BrainVoyager QX (Brain Innovation BV, The Netherlands). Nuisance signals (global signal, white matter and cerebro-spinal fluid signals and motion parameters) were regressed out from each data set. Before statistical analyses, individual functional data were co-registered to their own anatomical data and spatially normalized to Talairach space. Single-subject and group-level ICA was carried out respectively with the fastICA and the self-organizing group ICA [sogICA] algorithms [13]. For each subject, 40 independent components (corresponding to one sixth of the number of time points, see, e.g., Grecius et al., 2007) [17] were extracted. All single-subject component maps were then “clustered” at the group level, resulting in 40 single-group average maps that were visually inspected to recognize the main functional resting-state networks, and particularly, to select the DMN component. The sign-adjusted DMN components of all subjects were then submitted to a second-level multi-subject random effects analysis that treated the individual subject map values as random observations at each voxel. Single-group one-sample t-tests were used to analyze the whole-brain distribution of the DMN component in each group separately and the resulting t-maps were thresholded at *p* = 0.05 (Bonferroni corrected over the entire brain). An inclusive mask was also created from the union of the two single-group maps (patients with MwoA and HC) and used to define a new search volume for within-network between-group comparisons. The resulting statistical maps were overlaid on the standard “Colin-27” brain T1 template. To correct for multiple comparisons, regional effects were only accepted for clusters exceeding a minimum size determined with a non-parametric randomization approach. Namely, an initial voxel-level threshold was set to *p* = 0.01 (uncorrected) and a minimum cluster size was estimated after 1000 Montecarlo simulations that protected against false positive clusters up to 5%. Cluster-level correction is a very common and effective way to correct for multiple comparisons in fMRI statistical maps, including random-effects maps, obtained from RS-fMRI studies (see, e.g., Russo et al., 2012) [16]. Individual ICA z-scores for both groups were extracted from DMN clusters identified in the above analyses and used for linear correlation analyses with clinical parameters of disease severity and cognitive scores. ICA z-scores express the relative modulation of a given voxel by a specific ICA and hence reflect the amplitude of the correlated fluctuations within the corresponding functional connectivity network.

### VBM

Data were processed and examined using SPM8 software (Wellcome Trust Centre for Neuroimaging, London, UK; http://www.fil.ion.ucl.ac.uk/spm). VBM was implemented in the VBM8 toolbox (http://dbm.neuro.uni-jena.de/vbm.html) with default parameters incorporating the DARTEL toolbox, which was used to obtain a high-dimensional normalization protocol [18]. Images were bias-corrected, tissue-classified, and registered using linear (12-parameter affine) and non-linear transformations (warping) within a unified model. Subsequently, the warped gray matter (GM) segments were affine-transformed into Montreal Neurological Institute (MNI) space and were scaled by the Jacobian determinants of the deformations to account for the local compression and stretching that occurs as a consequence of the warping and affine transformation (modulated GM volumes). Finally, the modulated volumes were smoothed with a Gaussian kernel of 8-mm full-width at half maximum (FWHM). The GM volume maps were statistically analyzed using the general linear model based on Gaussian random field theory. Statistical analysis consisted of an analysis of covariance (ANCOVA) with total intracranial volume (TIV) and age as covariates of no interest. We assessed whole-brain regional differences, as well as differences over region of interest (ROI) based on the results of the whole-brain between groups RS-fMRI analysis. Statistical inference was performed at the voxel level, with both a family-wise error correction for multiple comparisons (*p* < 0.05) and an uncorrected threshold (*p* < 0.001; cluster size:100).

## Results

### Clinical and neuropsychological data

The groups (20 patients with MwoA and 20 HC) did not differ in age or male/female ratio (see Table 1 for further clinical details). Patients with MwoA and HC showed no significant differences in HDRS and HARS scores. Patients with MwoA did not show significant cognitive impairments as compared to published normative data (Table 2).

**Table 1:** Clinical characteristics of patients with MwoA and HC

**Parameter**	**Group**	**Mean ± SE**	**p-value**
**Gender**	MwoA	10 M / 10 F	n.s.
	HC	10 M / 10 F	n.s.
**Age (years)**	MwoA	28.15 ± 3.08	0.49
	HC	28.90 ± 3.63	
**Disease duration (years)**	MwoA	8.22 ± 2.04	
**Frequency (day/month)**	MwoA	6 ± 2.04	
**Side of attack**	MwoA	10R / 10 L	n.s.
**MIDAS**	MwoA	17.64 ± 5.25	
**HIT-6**	MwoA	60.21 ± 7.98	
**VAS of attack intensity**	MwoA	8.0 ± 1.65	

MwoA = patients with migraine without aura; HC = healthy controls; M = male; F = female; R = right; L = left; MIDAS = Migraine Disability Assessment Scale; HIT-6 = Headache Impact Test; VAS = Visual Analogue Scale.

**Table 2:** Neuropsychological evaluation in patients with MwoA

	**Mean ± SD**	Cut-off*
Education (years)	12.26 ±3.52	
**Global general cognition**		
MMSE	28.50 ± 1.30	>26/30
**Psychiatric symptoms**		
HARS	4.65 ± 2.50	<14
HDRS	4 ± 3.45	<10
**Neuropsychological test**		
TMT A	35 ± 6.58	≤ 94
TMT B	74.02 ± 12.2	≤ 283
WCST categories	105 ± 0.47	≥ 5.04
WCST err. perseveration	0.20 ± 0.38	≤ 5.6
WCST err. n. perseveration	0.22 ± 0.52	≤ 8.52
PF	28.16 ± 9.17	≥17.35
FAB	17.34 ± 5.49	≥12.03
Raven PM 47	28.71 ± 2.43	≥18.96

MMSE: mini mental state examination; HARS: Hamilton anxiety rating scale; HDRS: Hamilton depression rating scale; TMT A: Trail Making Test Part A; TMT B: Trail Making Test Part B; WCST: Wisconsin Card Sorting Test; PF: phonemic fluency; FAB: frontal assessment battery; Raven PM 47: Raven Standard Progressive Matrices.

*Values are relative to published normative data (see references for further details).

### RS- fMRI and VBM

**Figure 1:**
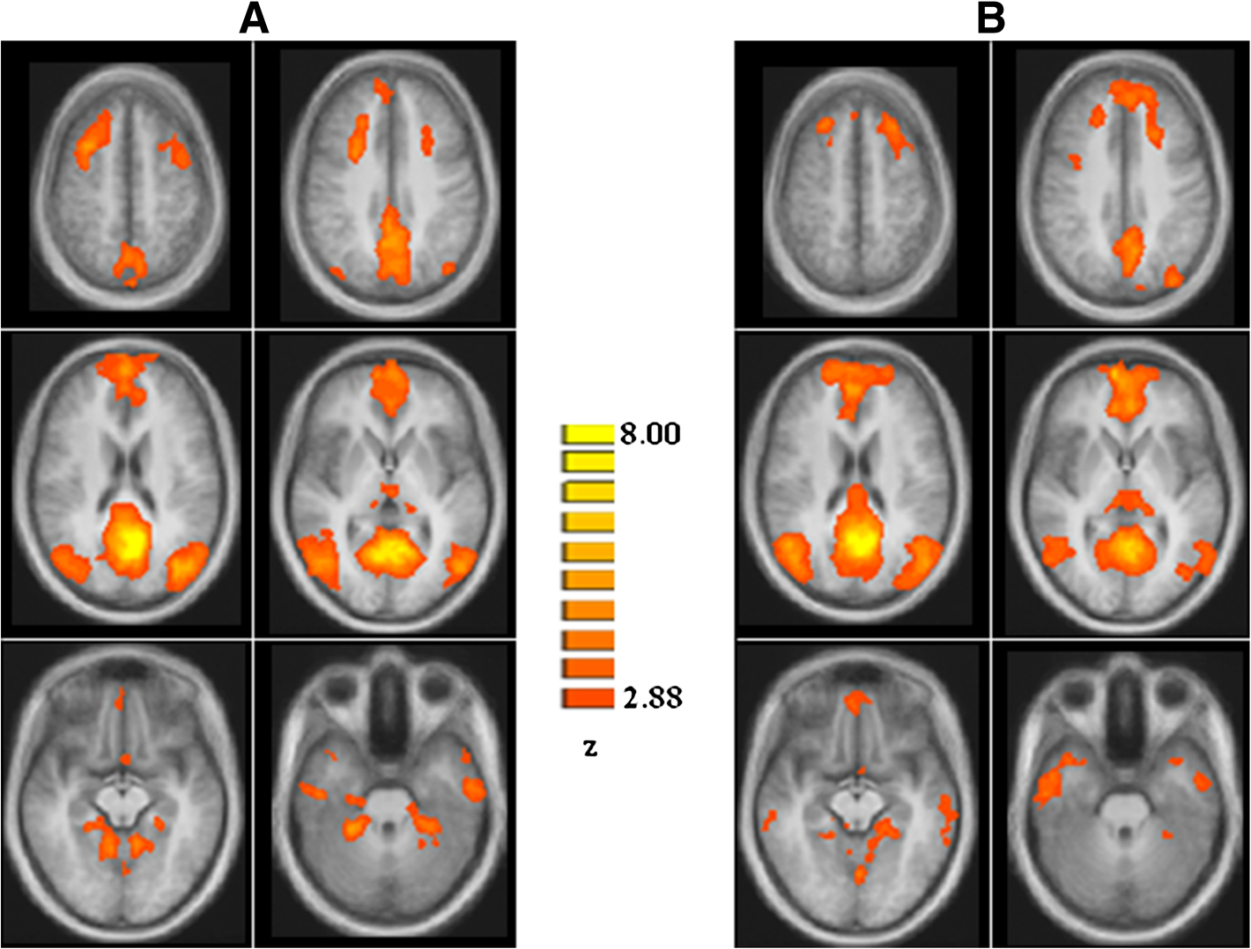
**Group level DMN connectivity in HC (A) and patients with MwoA (B) (*p*** **< 0.05, cluster-level corrected) HC: healthy controls; MwoA: migraine without aura.**

**Figure 2:**
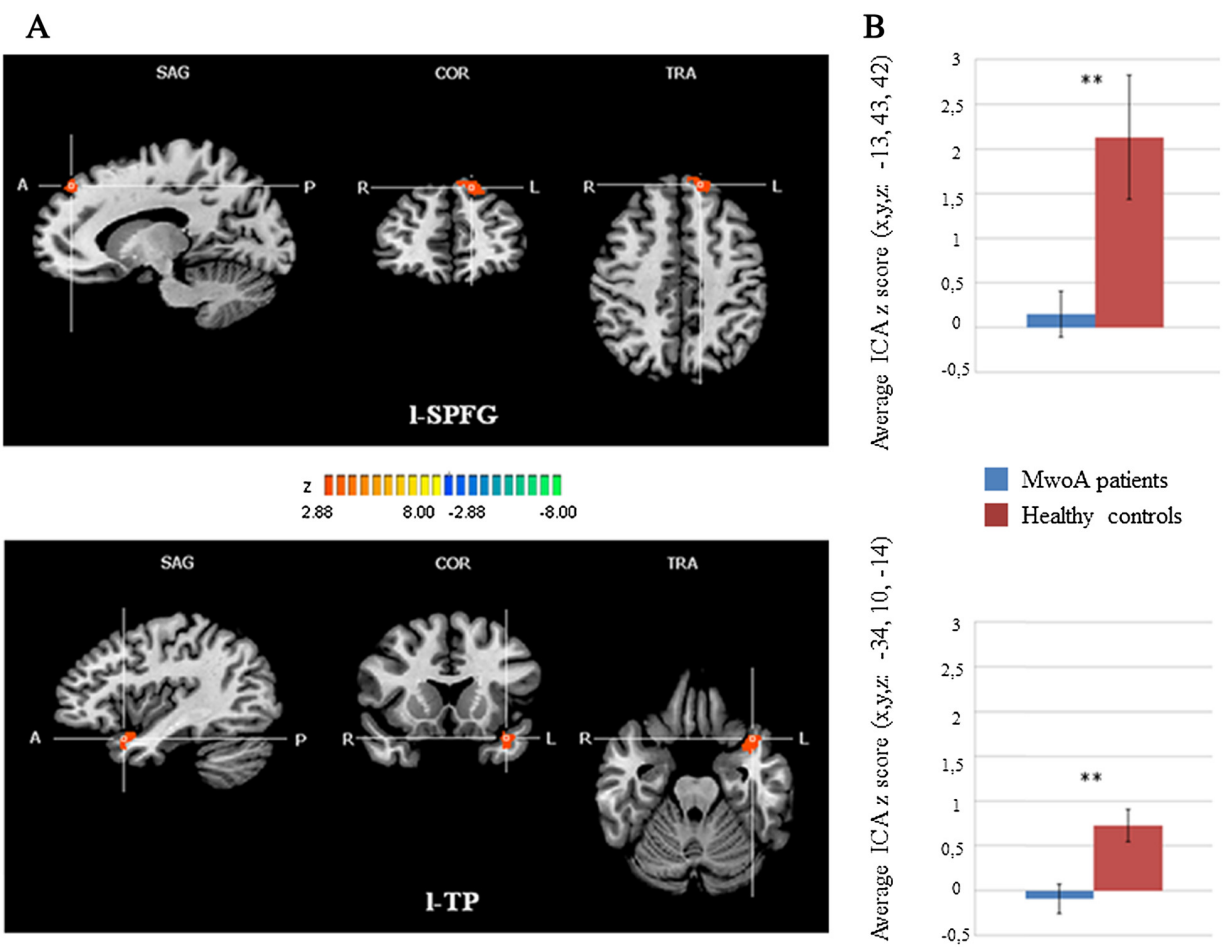
**Statistically significant differences within the DMN between patients with MwoA and HC groups. A)** T-map of statistically significant differences within the DMN between patients with MwoA and HC groups (*p* < 0.05, cluster-level corrected) overlaid on the standard “Colin-27” brain T1 template. Talairach coordinates (x,y,z): top: right l-SPFG = −13, 43, 42; bottom: right l-TP = −34, 10, -14. **B)** Bar graphs of the ROI-averaged ICA z-scores (±SD) for patients with MwoA and HC groups. Top: l-SPFG (MwoA patients: 0.15 ± 0.63; HC: 2.12 ± 0.43; p = 0.01). Bottom: l-TP (MwoA patients: -0.09 ± 0.55; HC: 0.72 ± 0.29; *p* = 0.003). DMN: default mode network, MwoA: migraine without aura, HC: healthy controls, ICA: independent component analysis, l-SPFG: left superior prefrontal gyrus; l-TP: left temporal pole.

As illustrated in Figure 1, each group exhibited a DMN connectivity pattern consistent with prior reports, encompassing medial and inferior prefrontal cortices, temporal lobe areas, anterior and posterior cingulate cortices, precuneus and cerebellar areas [6-8]. The two-sample t-tests revealed significant group differences in the left superior prefrontal gyrus (l-SPFG) (Talairach coordinates x,y,z: -13, 43, 42; Brodmann area 8) and in the left temporal pole (l-TP) (Talairach coordinates x,y,z: -34, 10, -14; Brodmann area 38), indicating that these regions had reduced component time course-related activity in patients with MwoA compared to HC (Figure 2A and 2B). Post-hoc correlation analyses revealed that individual ROI averaged ICA scores in the l-SPFG and l-TP were not correlated neither with clinical parameters of disease severity (i.e. duration, frequency, VAS, MIDAS and HIT-6 scores) nor with single cognitive tests scores. There were no differences in global GM, white matter (WM) or cerebro-spinal fluid (CSF) volumes between groups (GM: MwoA patients = 684.53 mm3 ± 77.80 mm3; HC = 688.32 mm3 ± 71.12 mm3; *p* = 0.72; WM: MwoA patients = 516.03 mm3 ± 59.60 mm3; HC = 451.33 mm3 ± 85.88 mm3; *p* = 0.61; CSF: MwoA patients = 203.67 mm3 ± 26.17 mm3; HC = 207.89 mm3 ± 48.31 mm3; *p* = 0.64; total atrophy: MwoA patients = 1419.11 mm3 ± 123.33 mm3; HC = 1347.08 mm3 ±179.21 mm3; *p* = 0.82). Moreover, both whole-brain and ROI-based analyses of regional volumes did not reveal any significant differences in local GM between patients with MwoA and HC, using a significance level of *p* ≤ 0.05, FWE-corrected for multiple comparisons.

## Discussion

The present RS-fMRI study was designed to assess the functional integrity of DMN in patients with MwoA. Our findings demonstrate a reduced functional connectivity within the prefrontal and temporal cortices of the DMN in patients with MwoA during the interictal period. This altered functional connectivity was independent of structural abnormalities and not related to clinical or cognitive features of migraineurs. The DMN is a network highly relevant for cognitive processes and influences behavior in response to the environment in a predictive manner [7,8]. In other terms, DMN represents a neural network related to individual stressful experiences and coping strategies to promote adaptation (i.e. allostasis) [19,20]. This is done requiring most energy of brain baseline metabolic rate due to elevated levels of aerobic glycolysis required by the DMN [21]. Previous RS-fMRI studies investigating brain functional connectivity in patients suffering from different chronic pain conditions have already shown a dramatic alteration of DMN connectivity, suggesting that pain has a widespread impact on overall brain function, modifying brain dynamics beyond pain perception [22,23]. Although several RS-fMRI studies, using different methodological approaches, have disclosed diffuse alterations in different brain areas and networks in patients with migraine [10-12,24,25], only a few of them have specifically investigated DMN integrity [10-12]. Xue and colleagues [10] have demonstrated an aberrant connectivity within the salience and executive networks in patients with MwoA; whereas DMN did not show any significant intra-network changes between patients with MwoA and HC. Nevertheless, an increased intrinsic DMN connectivity to brain regions outside the usual boundaries of this network (i.e. right insula) was reported. In another RS-fMRI study [11], the same group, using amplitude of low-frequency fluctuation and ROI-based functional connectivity analyses, has demonstrated a reduced DMN connectivity in left anterior cingulate cortex, bilateral prefrontal cortex and right thalamus. Furthermore, a significant decrease in regional homogeneity values has been observed in several brain areas involved in DMN in patients with MwoA [12]. DMN functional changes were negatively correlated only with disease duration [10-12]. These conflicting data may be explained by the small sample size, patients clinical heterogeneity, and lack of consistent methodological approach. Furthermore, it is noteworthy that in those studies the cognitive profile of patients with migraine was not investigated, then behavioral correlates of the observed functional abnormalities are still unclear. In the present study, to specifically address this issue, we have performed a correlation analysis between clinical, cognitive and functional data, and we did not find any significant association. This is not surprising, considering our previous study [16] showing no correlation between executive network changes and neuropsychological data in patients with MwoA. Thus, taken together, our findings may suggest a possible alternative behavioral correlation of resting-state connectivity changes. One possibility is that the observed DMN dysfunction could underlie or be related to a maladaptive brain response to repeated stress [19,20] which seems to characterize patients with migraine [4,5]. Indeed, according to recent studies, recurrent migraine attacks alter both functional and structural brain connectivity [14], and these changes may disrupt mechanisms of stress response [4,5]. When behavioral or physiological stressors are frequent or severe, allostatic responses can become maladaptive, leading, in a vicious cycle, to further allostatic load. Moreover, due to a high energetic demand, the observed DMN dysfunction may be associated with an impaired brain energy metabolism which has been demonstrated in previous MR spectroscopy studies in patients with migraine [26], likely due to an imbalance between ATP production and ATP use. In support of this notion, metabolic enhancers, such as riboflavin and coenzyme Q10 (both with a well-defined role in ATP generation), have shown effects in migraine prophylaxis [27]. In the present study, we identified two core regions of DMN [8], namely prefrontal and temporal areas, showing reduced functional connectivity in patients with MwoA. These areas have been demonstrated to be crucially involved in sensory-discriminative, cognitive and integrative pain functions within the so-called “neurolimbic pain network” [28]. In details, prefrontal cortex plays a specific role in mediating the attenuation of pain perception via cognitive control mechanisms [29,30] whereas temporal cortex is involved in affective response to pain experience and its activation has been demonstrated both during pain experience [31] and migraine attacks [32]. Moreover, recent studies have reported both cortical abnormalities and microstructural changes of these regions in migraineurs [33-35]. However, in the present study DMN connectivity disruption was detected in the absence of significant GM changes, possibly implying that functional changes may precede GM structural abnormalities. A few limitations of the current study should be considered. First, our methodological approach using ICA allows to evaluate functional interactions between brain areas but it does not provide information regarding causality and, consequently, it is still unclear whether functional changes are cause or consequence of repetitive migraine attacks. Second, we have studied a relatively small number of patients and further studies are needed to confirm our findings. Finally, the relationship between DMN functional changes and maladaptive brain response could be considered as a working hypothesis emerged from our work and future RS-fMRI studies are needed to further elucidate this potential correlation.

## Conclusions

We believe that DMN connectivity changes may represent an early migraine biomarker, probably related to a maladaptive brain response. Future studies should examine other cortical resting-state networks and longitudinal studies are needed to evaluate the possibility that this modern neuroimaging approach can lead to the identification of different categories of patients or different timing of selective networks involvement.

## Abbreviations

MwoA: Migraine without aura; HC: Healthy controls; RS: Resting-state; fMRI: Functional magnetic resonance; RSNs: Resting-state networks; ICA: Independent component approach; VBM: Voxel based morphometry; l-SPFG: Left superior prefrontal gyrus; l-TP: Left temporal pole; MIDAS: Migraine disability assessment scale; HIT-6: Headache impact test; VAS: Visual analogic scale; HDRS: Hamilton depression rating scale; HARS: Hamilton anxiety rating scale; MNI: Montreal neurological institute; FWHM: Full-width at half maximum; ANCOVA: Analysis of covariance; TIV: Total intracranial volume; TR: Repetition time; TI: Inversion time; TE: Echo time; ROI: Region of interest; GM: Gray matter; WM: White matter; CSF: Cerebro-spinal fluid; FWE: Familywise error rate; ATP: Adenosine triphosphate.

## Competing interests

The authors confirm that there are no conflicts of interest.

## Authors’ contributions

AT: experimental design, image data analysis, results interpretation, manuscript drafting; AR: literature review, experimental design, results interpretation, manuscript drafting; AG: clinical data analysis, results interpretation and manuscript revision; FC: clinical data analysis, manuscript revision; DC: image data analysis, results interpretation; MDS: neuropsychological data acquisition, results interpretation; SC: image data analysis, results interpretation, manuscript revision; MC: image data acquisition, results interpretation; FE: image data analysis, results interpretation and manuscript drafting and revision; GT: experimental design, results interpretation, manuscript revision. All authors read and approved the final manuscript.
